# Remote ischemic conditioning during primary percutaneous coronary intervention in patients with ST-segment elevation myocardial infarction: a systematic review and meta-analysis

**DOI:** 10.1186/s13019-019-0834-x

**Published:** 2019-01-21

**Authors:** Ren Gong, Yan-Qing Wu

**Affiliations:** Department of Cardiovascular Medicine, The Second Affiliated Hospital of Nanchang University, No. 1 Minde Road, Nanchang, 330006 Jiangxi China

**Keywords:** Remote ischemic conditioning, Ischemic conditioning, Myocardial infarction, Percutaneous coronary intervention, Meta-analysis

## Abstract

**Objective:**

This systematic review was designed to evaluate the efficacy of remote ischemic conditioning (RIC) with primary percutaneous coronary intervention (PCI) versus primary PCI alone for ST-segment elevation myocardial infarction (STEMI).

**Search strategy:**

Computerized search for trials from PubMed, EMBASE, CENTRAL and Cochrane Database of Systematic Reviews databases.

**Selection criteria:**

Trials investigating RIC plus primary PCI (group A) versus primary PCI alone (group B).

**Outcome measures:**

Myocardial enzyme levels; left ventricular ejection fraction (LVEF); major adverse cardiac and cerebrovascular events (MACCEs); TIMI flow grade III; myocardial salvage index or infarct size per patients.

**Results:**

In all, 14 studies involving 3165 subjects were included. There was a significant association of myocardial edema levels, myocardial salvage index and incidence of MACCEs in group A compared with group B (myocardial edema levels: SMD = − 0.36, 95% CI (− 0.59, − 0.13); myocardial salvage index: MD = 0.06, 95% CI (0.02, 0.10); MACCE: OR = 0.70, 95% CI (0.57, 0.85)). With regard to infarct size, TIMI flow grade III and LVEF, group A appeared to be equivalent with group B (infarct size: MD = − 1.67, 95% CI (− 3.46, 0.11); TIMI flow grade III: OR = 1.04, 95% CI (0.71, 1.52); LVEF: MD = 0.74, 95% CI (− 0.80, 2.28)).

**Conclusion:**

RIC was associated with lower myocardial edema levels, myocardial salvage index and incidence of MACCE, while non-significant beneficial effect on infarct size, TIMI flow grade III or LVEF. These findings suggest that RIC is a promising adjunctive treatment to PCI for the prevention of reperfusion injury in STEMI patients.

## Introduction

Percutaneous coronary intervention (PCI), with its full and lasting opening infarct-related artery, has become one of the most effective means for treating ST-segment elevation myocardial infarction (STEMI) [1]. However, ischemia-reperfusion injury can cause myocardial necrosis or no reflux phenomenon and affect the short-term and long-term prognosis of patients [2, 3]. How to reduce myocardial ischemia and reperfusion-induced injury has become our focus.

Ischemic preconditioning refers to the application of a shorter, non-fatal, ischemia-reperfusion treatment before a longer ischemic injury to reduce long-term ischemic injury to tissues. A large number of experimental and clinical studies have confirmed the protective effect of ischemic preconditioning and considered it to be the strongest and most effective cardioprotective measure currently available [4, 5]. In 2005, Staat et al. first reported another endogenous cardioprotective mechanism [6]. During percutaneous transluminal coronary angioplasty, balloon inflation and deflation were used to achieve interruption and recovery of blood flow. This method can reduce the myocardial infarct size, protect coronary artery endothelial function and reduce the ischemic myocardium inflammatory response and other cardiac protection, similar to ischemic preconditioning, and this method is called ischemic postconditioning [7]. Although myocardial ischemic preconditioning and postconditioning can produce cardio-protective effects and reduce myocardial ischemia-reperfusion injury, it is itself traumatic and presents a variety of potential risks, which reduces its clinical feasibility. The remote organ ischemic preconditioning and post-treatment have the characteristics of simple implementation, and less trauma, has and have certain clinical feasibility. Remote ischemic conditioning (RIC) is easy to operate and has few side effects. It protects important vital organs through ischemic preconditioning of organs. Therefore, it will be of great clinical value to conduct in-depth research on it. The purpose of this meta-analysis was to evaluate the effect of randomized controlled trials of RIC in STEMI patients undergoing elective PCI.

## Materials and methods

This meta-analysis was performed according to the Cochrane Handbook for Systematic Reviews of Interventions [8] and presented based on the Preferred Reporting Items for Systematic Reviews and Meta-analyses guidelines [9].

### Search strategy

We conducted a systematic search of the PubMed, EMBASE, Cochrane Central Register of Clinical Trials (CENTRAL) and Cochrane Database of Systematic Reviews, using the MeSH terms and free key words “ischemic conditioning”, “remote conditioning”, “remote ischemic conditioning”, “myocardial infarction”, “STEMI” or “percutaneous coronary intervention” from their dates of inception to March, 2018, and identified all potentially relevant articles. There was no language restriction. We also searched the reference lists of the full-text papers and reviewed studies from all of the relevant publications to identify any omitted studies.

### Inclusion criteria

Inclusion criteria were articles relating to: 1) STEMI patients undergoing primary PCI**;** 2) trials focused on comparing RIC with no conditioning (control) group; 3) randomized controlled trials (RCTs). Articles with the following exclusion criteria were eliminated:1) STEMI patients undergoing thrombolysis; 2) unreported myocardial parameters or clinical outcomes; 3) case reports, or observational studies; 4) duplicated previous literature.

### Outcomes measures

Myocardial enzyme levels, including creatine kinase isoenzyme MB (CK-MB) (peak or area under the curve), troponins (peak or area under the curve); left ventricular ejection fraction (LVEF%); major adverse cardiac and cerebrovascular events (MACCE); TIMI flow grade III; myocardial salvage index and infarct size. At least one of the outcome measures mentioned above must be reported in the included articles.

### Risk-of-bias assessments

The risk of bias in each included study was evaluated based on Cochrane handbook version 5.1.0 for Systematic Reviews by Cochrane Collaboration. Study quality was evaluated including random sequence generation, allocation concealment, blinding of participants and personnel, blinding of outcome assessment, incomplete outcome data, selective reporting and other biases. Each entry was then classified as “low risk”, “unclear risk” and “high risk”.

### Data selection and extraction

Trials identified through the search activities described above were each assigned to a review topic (or topics). Data extracted from the review were entered into Thomson Research Software (EndNote X4), and checked for accuracy. When information regarding any of the above was unclear, original reports were examined for further details. “Included”, “pending”, “excluded (reason)” were indicated into the “notes” column, and “pending” reports were retraced from the references.

A self-designed data extraction form was used to independently extract contents by two researchers including lead author, year of publication, participant characteristics, RIC protocol, and outcome measures. Literature screening, quality evaluation and data extraction were carried out by two reviewers. In case of disagreement, a third investigator helped resolve the disagreement or through discussion.

### Statistical analysis

Review Manager Software (RevMan5.3 offered by the Cochrane Collaboration) was used for statistical analysis. Mean difference (MD) and its 95% confidence interval (CI) presented the result of meta-analysis for continuous outcomes. When different scales were used, the standardized MD (SMD) was calculated. Odds ratios (OR) and its 95% CI were used for binary data meta-analysis of effect size. Chi-square test was used to assess significance of heterogeneity, and the degree of heterogeneity was then examined using the I^2^ statistic. The fixed-effects model was used if the assessment of heterogeneity was not significant (p > 0.1,I^2^ ≤ 50%). If the source of heterogeneity was uncertain, the random-effects model was used for analysis.

## Results

### Study selection

**Fig. 1:**
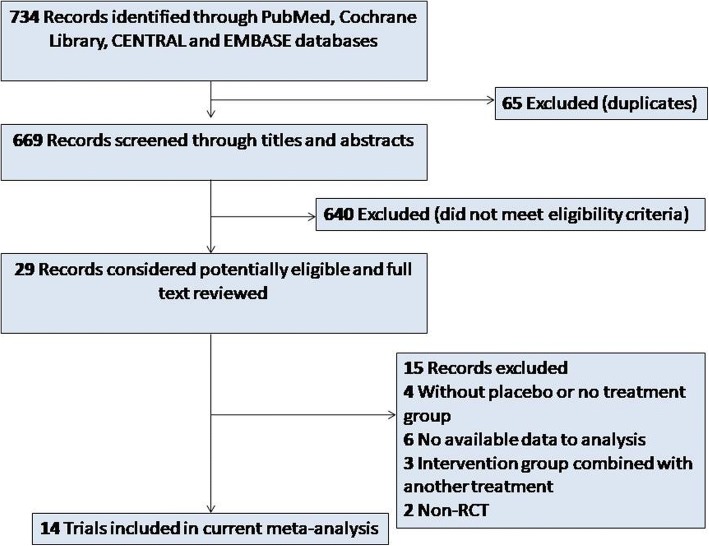
The flow diagram of study searching strategy

A total of 734 articles were retrieved. After 65 duplicate papers were removed, 640 irrelevant citations were excluded based on review of titles and abstracts. Intensive full-text review of the 29 included articles further eliminated 15 articles. Finally, a total of 14 studies [10–23] published between 2010and 2018 were assessed for eligibility in the meta-analysis (Fig. 1).

### Quality assessment

**Fig. 2:**
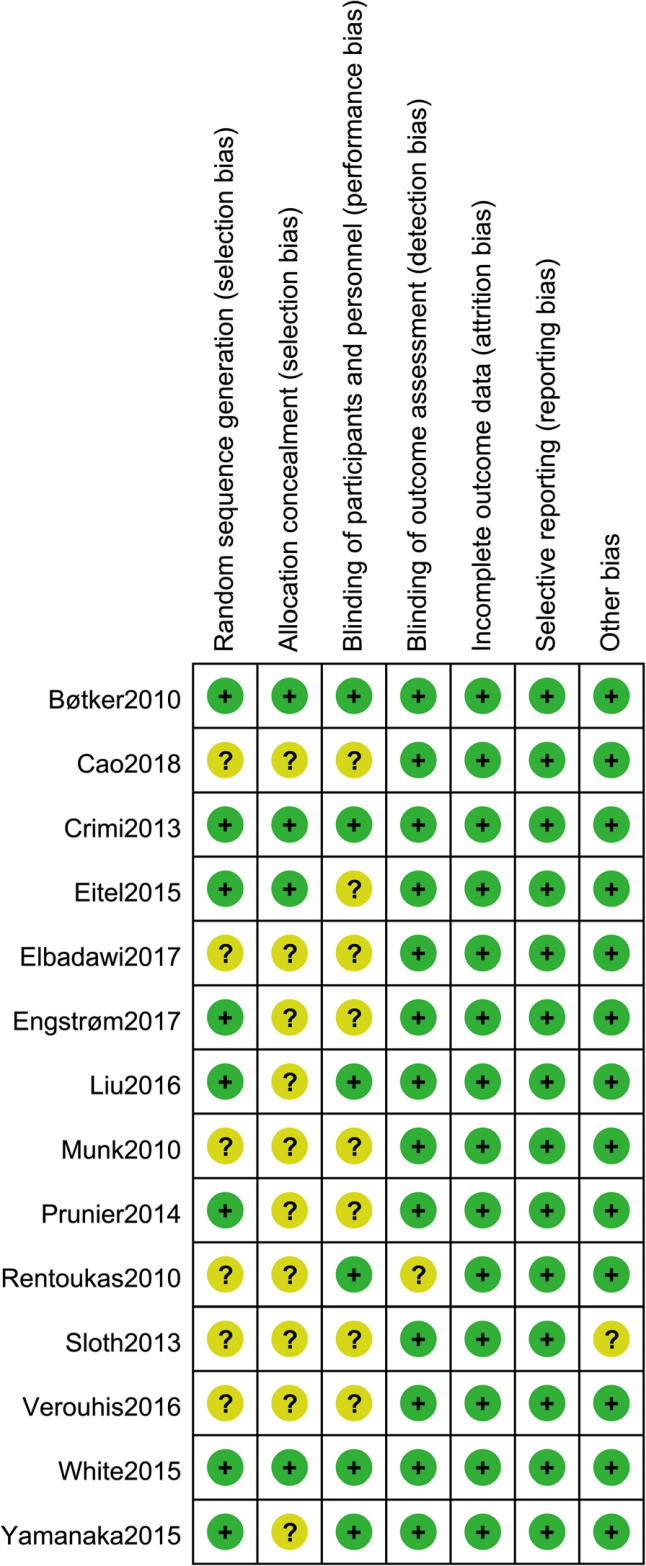
Quality assessment summary of the included studies

**Fig. 3:**
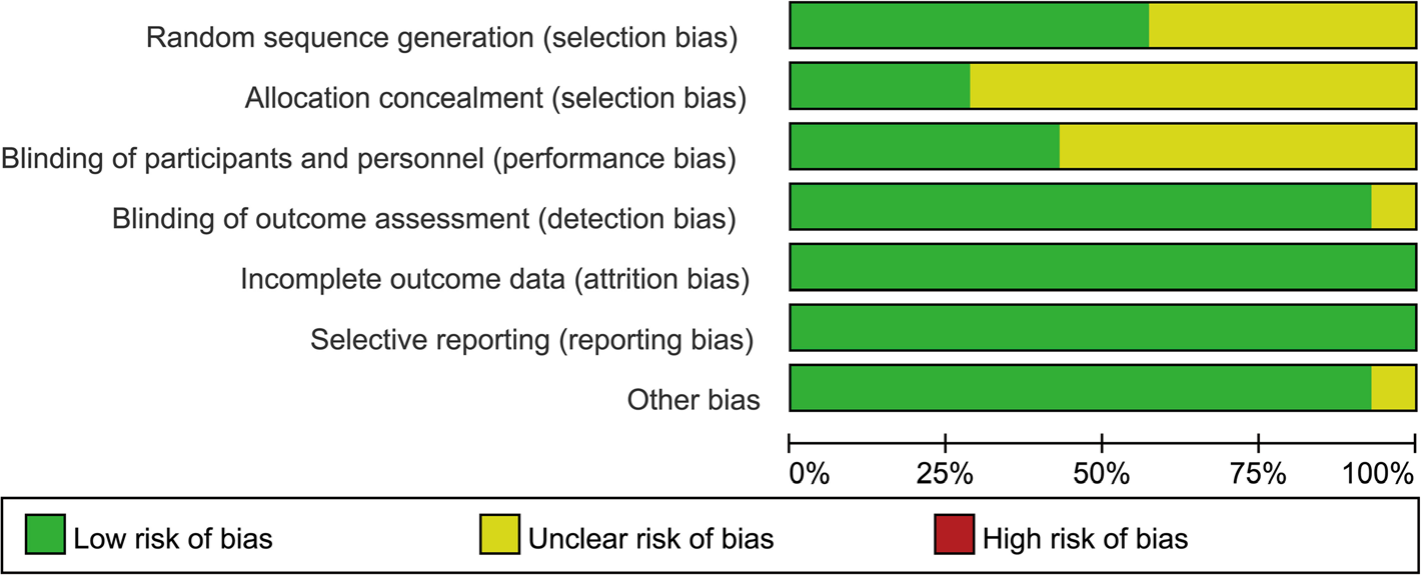
Methodological quality assessment of each included study “+”, low risk of bias; “-”, high risk of bias; “?”, unclear risk of bias

Eight studies [10, 12, 13, 15, 16, 18, 22, 23] used random sequence generation by computer-generated random sequence, while the remaining 6 studies were just reported as randomized trials and offered no description on randomization methods. Four trials [10, 12, 13, 22] allocated patient concealment by sealed envelope. Blinding methods were used in 6 trials [10, 12, 16, 19, 22, 23]. Blinding of outcome assessment independent of treatment was used in most trials except 1 trial [19]. None of the included studies had incomplete report or selective report. Overall, 6 studies [10, 12, 13, 16, 22, 23] were of high methodological quality, and the rest were of moderate quality. A summary of the quality assessment results for all of the trials is shown in Fig. 2 and Fig. 3.

### Characteristics of study selection

Totally, 3165 STEMI patients were included in this meta-analysis, 1585 receiving RIC plus primary PCI (group A), and 1580 receiving primary PCI alone (group B). Studies included STEMI patients with symptom onset < 24 h, TIMI flow grade > I in 3 trials [12, 15, 22]. Their mean age ranged from 50 to 80 years, and the sample size ranged from 35 to 1234. RIC protocols were similar among most of the studies. The characteristics of the included studies are depicted in Table 1.

**Table 1:** Characteristics of included studies

Author, year	Inclusion Criteria, TIMI flow grade	Samplesize, gender M/F		Mean age, years (SD)		RIC Protocol				Outcome measures
		RIC + PCI	PCI	RIC + PCI	PCI	Sit of cond	Timing of cond.	Duration of cond.(min)	Tourniquet pressure (mmHg)	
Bøtker,2010 [10]	STEMI, symptom onset< 12 h;TIMI flow grade 0-III	96/30	94/31	62(12)	63(11)	Upper limb	Pre	40	200	①②③④⑥
Cao,2018 [11]	STEMI, symptom onset< 6 h; NR	29/7	40/4	59(13)	59(10)	Upper limb	Post	40	200	①④⑤
Crimi,2013 [12]	Anterior STEMI, symptom onset< 6 h; TIMI flow grade 0-I	41/7	43/5	61(11)	56(11)	Lower limb	Post	30	200	①③④⑤⑥
Eitel,2015 [13]	STEMI, symptom onset< 12 h; TIMI flow grade 0-III	169/63	165/67	65(10)	65(9)	Upper limb	Pre + Post	30	200	②③⑤⑥
Elbadawi, 2017 [14]	Anterior STEMI, symptom onset< 12 h; NR	5/25	5/25	53(8)	50(7)	Lower limb	Post	30	200	①④⑤⑥
Engstrøm, 2017 [15]	STEMI, symptom onset< 12 h; TIMI flow grade 0-I	489/128	486/131	63(11)	62(12)	Lower limb	Post	20	200	②③⑤⑥
Liu,2016 [16]	STEMI, symptom onset< 12 h; TIMI flow grade 0-III	45/14	49/11	62(12)	63(12)	Upper limb	Pre	40	200	③⑤⑥
Munk,2010 [17]	STEMI, symptom onset< 12 h; NR	28/95	26/93	62(11)	62(11)	Upper limb	Pre	40	200	⑤
Prunier,2014 [18]	STEMI, symptom onset< 6 h; NR	14/4	13/4	66(16)	62(14)	Upper limb	Pre	30	200	①
Rentoukas, 2010 [19]	STEMI, symptom onset< 6 h; NR	20/13	18/12	63(11)	61(11)	Upper limb	Pre	24	20 mm Hgabove systolic arterial pressure	①④
Sloth,2013 [20]	STEMI, symptom onset< 12 h; TIMI flow grade 0-III	126	125	NR	NR	Upper limb	Pre	40	200	⑥
Verouhis, 2016 [21]	Anterior STEMI, symptom onset< 6 h; TIMI flow grade 0-III	44/3	44/2	61(8)	61(5)	Lower limb	Pre	70	200	①②③④⑤⑥
White,2015 [22]	STEMI, symptom onset< 12 h; TIMI flow grade 0-I	37/6	30/10	57(10)	60(11)	Upper limb	Pre	40	200	①②③④⑤
Yamanaka, 2015 [23]	STEMI, symptom onset< 24 h; NR	34/13	36/11	67(12)	67(15)	Upper limb	Pre	30	200	①⑤⑥

RIC: remote ischemic conditioning; PCI: percutaneous coronary intervention; STEMI: ST-segment elevation myocardial infarction; NR: not report; Outcome measures:①myocardial enzyme levels; ②myocardialsalvage index; ③infarct size; ④TIMI flow grade III; ⑤left ventricularejection fraction (LVEF); ⑥major adverse cardiac and cerebrovascular events (MACCE)

## Outcomes and synthesis of results

### Myocardial enzyme levels

**Fig. 4:**
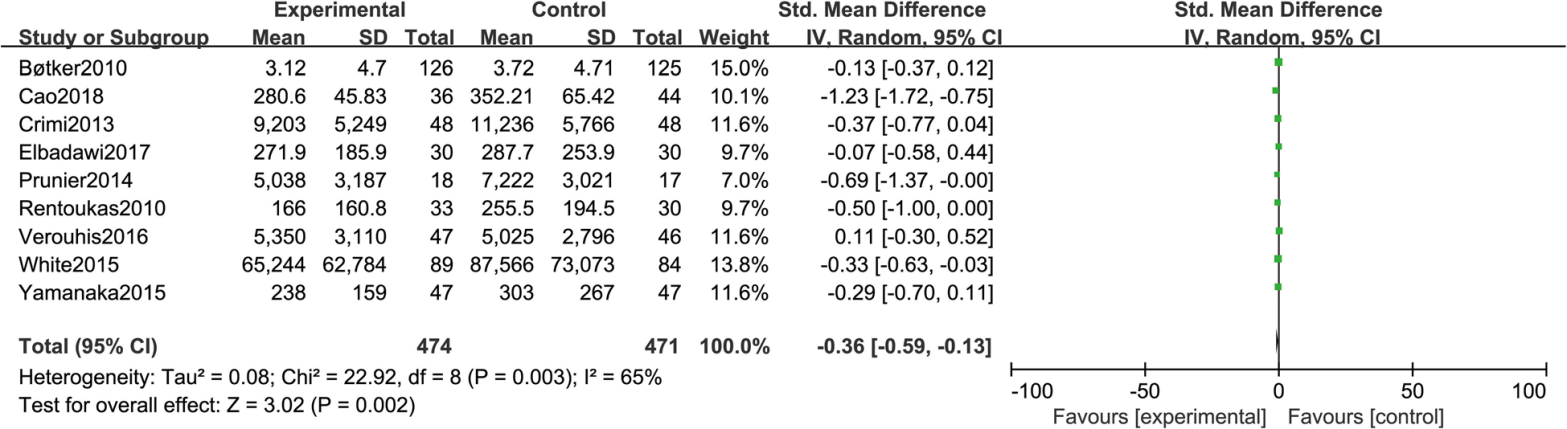
Comparison of myocardial enzyme levels between group A and group B

Nine studies [10–12, 14, 18, 19, 21–23]reported myocardial enzyme levels, including a total of 945 patients (474 patients in group A and 471 in group B). There was statistical between-study heterogeneity in SMD of studies (*P* = 0.003, I^2^ = 65%); therefore, we used the random effects model for merging. The pooled estimates of effect sizes showed that the difference of myocardial enzyme levels between the two groups was statistically significant (SMD = − 0.36, 95% CI (− 0.59, − 0.13), *P* = 0.002) (Fig. 4).

### Myocardial salvage index

**Fig. 5:**
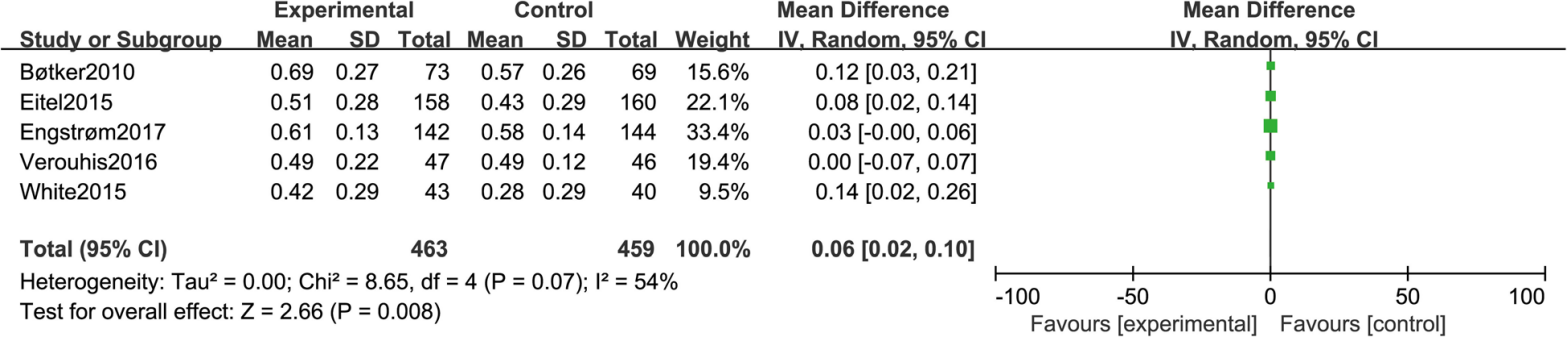
Comparison of myocardial salvage index between group A and group B

Five studies [10, 13, 15, 21, 22] reported myocardial salvage index, including a total of 922 patients (463 in group A and 459 in group B). There was statistical between-study heterogeneity in SMD of studies (*P* = 0.07, I^2^ = 54%); therefore, we used the random effects model for merging. The pooled estimates of effect sizes showed that the difference of myocardial salvage index between the two groups was statistically significant (MD = 0.06, 95% CI (0.02, 0.10), *P* = 0.008) (Fig. 5).

### Infarct size

**Fig. 6:**
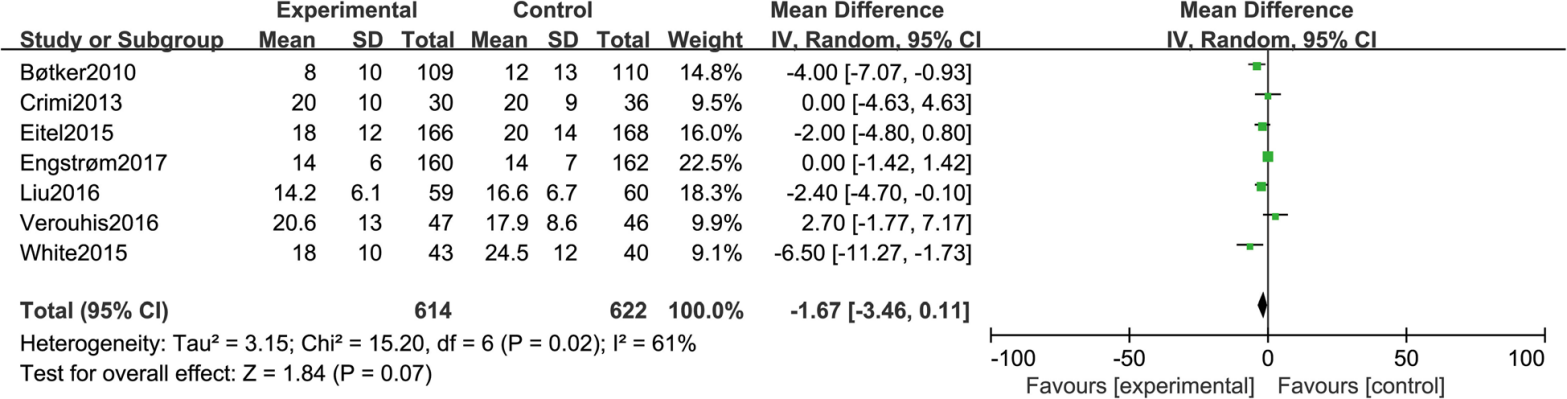
Comparison of infarct size between group A and group B

Seven studies [10, 12, 13, 15, 16, 21, 22] reported infarct size, including a total of 1236 patients (614 in group A and 622 in group B). There was statistical between-study heterogeneity in SMD of studies (*P* = 0.02, I^2^ = 61%); therefore, we used the random effects model for merging. The pooled estimates of effect sizes showed that the difference of infarct size between the two groups was not statistically significant (MD = − 1.67, 95% CI (− 3.46, 0.11), *P* = 0.07) (Fig. 6).

### TIMI flow grade III

**Fig. 7:**
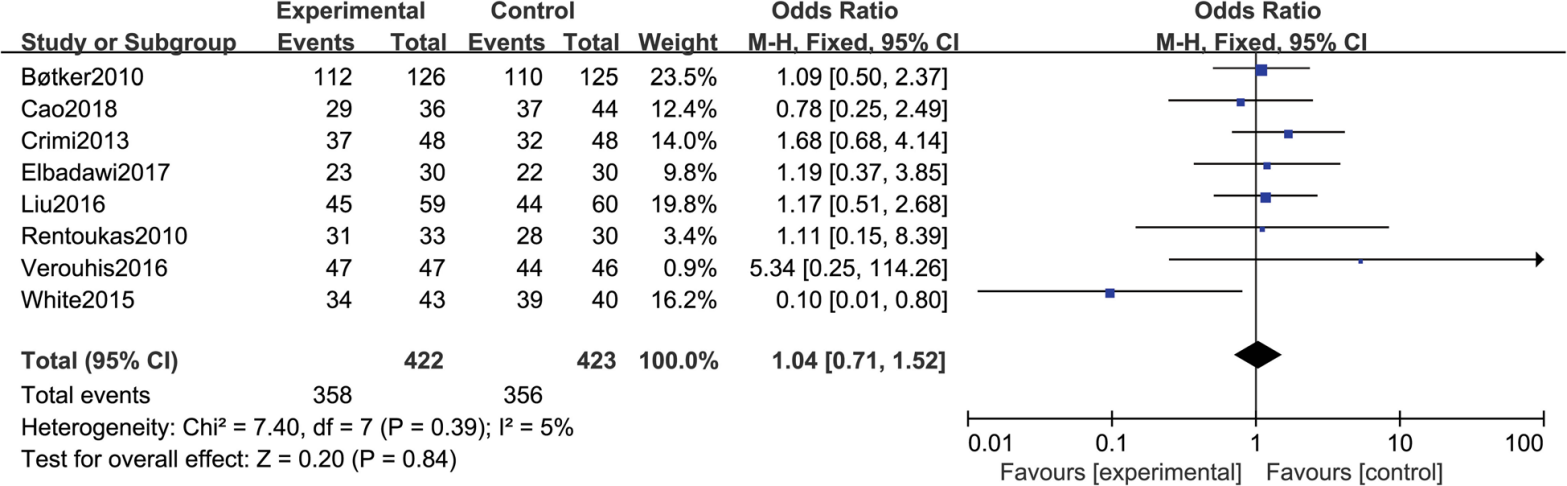
Comparison of TIMI flow grade III between group A and group B

Eight studies [10–12, 14, 16, 19, 21, 22] reported TIMI flow grade III, including a total of 845 patients (422 in group A and 423 in group B). There was no statistical between-study heterogeneity in OR of studies (*P* = 0.39, I^2^ = 5%); therefore, we used the fixed effects model for merging. The pooled estimates of effect sizes showed that the difference of TIMI flow grade III between the two groups was not statistically significant (OR = 1.04, 95% CI (0.71, 1.52), *P* = 0.84) (Fig. 7).

### LVEF

**Fig. 8:**
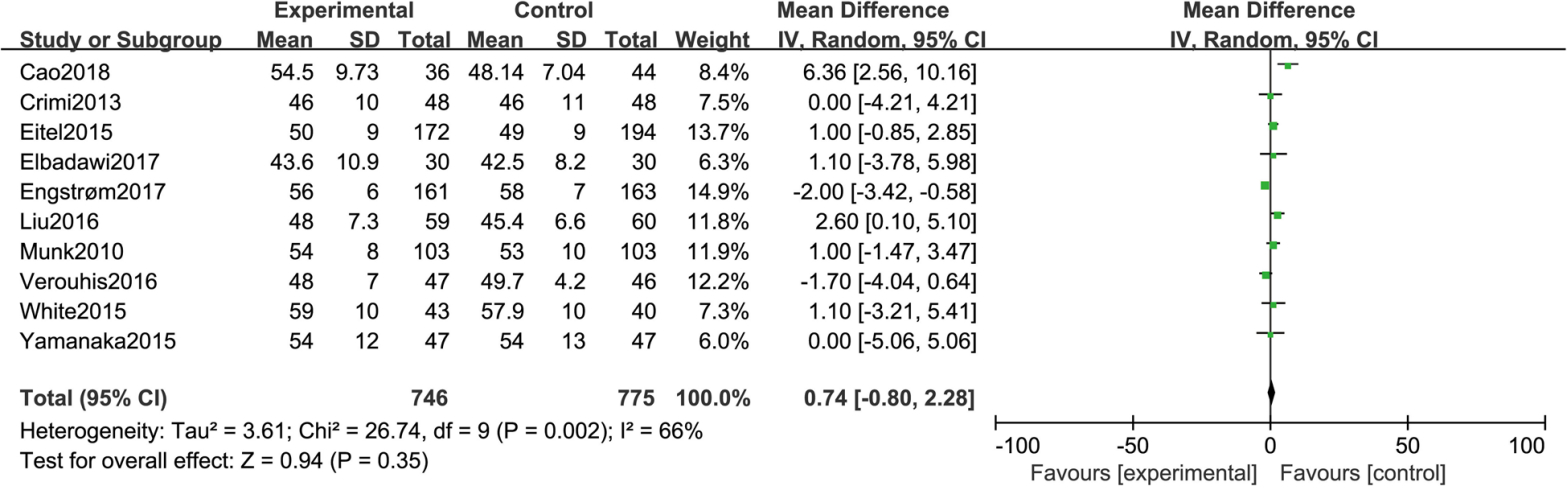
Comparison of LVEF between group A and group B

Ten studies [11–17, 21–23] reported LVEF, including a total of 1521 patients (746 in group A and 775 in group B). There was statistical between-study heterogeneity in SMD of studies (*P* = 0.002, I^2^ = 66%); therefore, we used the random effects model for merging. The pooled estimates of effect sizes showed no statistically significant difference in LVEF between the two groups(MD = 0.74, 95% CI (− 0.80, 2.28), *P* = 0.35) (Fig. 8).

### MACCE

**Fig. 9:**
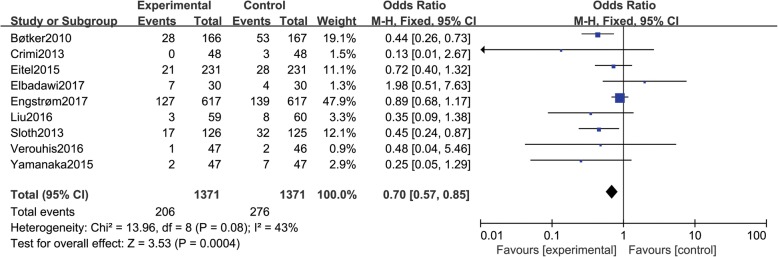
Comparison of MACCEs between group A and group B

Nine studies [10, 12–16, 20, 21, 23] reported MACCE, including a total of 2742 patients (1371 in group A and 1371 in group B). There was no statistical between-study heterogeneity in OR of studies (*P* = 0.08, I^2^ = 43%); therefore, we used the fixed effects model for merging. The pooled estimates of effect sizes showed t statistically significant difference in MACCE between the two groups (OR = 0.70, 95% CI (0.57, 0.85), *P* = 0.0004) (Fig. 9).

## Discussion

The results of this meta-analysis showed that RIC in patients undergoingPCI for STEMI was significantly associated with lower myocardial edema levels, myocardial salvage index and incidence of MACCEs. No benefit was demonstrable ininfarct size, TIMI flow grade III or left ventricular ejection fraction (LVEF).

PCI is the primary measure to treat acute STEMI clinically. Although acute restoration of myocardial blood flow is overall beneficial, the procedure in itself may jeopardize the myocardium. Patients receiving such treatment will risk sustaining reperfusion injury, which could potentially increase the final myocardial infarct size. Therefore, how to prevent and reduce reperfusion injury is important for myocardial protection and prognosis of patients. Remote ischemic pre- and post-conditioning, induced by repeated brief periods of limb ischemia before index ischemia, are recommended therapy for cardioprotection and myocardial injury prevention. RIC can provide a variety of protections against ischemic myocardium by limiting the myocardial infarct size and edema, reducing reperfusion-induced apoptosis, improving ventricular function (increased left ventricular diastolic pressure and decreased right ventricular diastolic pressure), and regulating myocardial remodeling after infarction [24]. Despite its potential clinical significance, the exact mechanisms of RIC have not yet been elucidated. The humoral and neural signaling pathways, as well as the systemic inflammatory response, may work in combination [25, 26].

Most included trials in this study chose a segment of the distal upper extremity or lower extremity prior to prolonged ischemia of the heart, and then inflate the cuff for 5 min (pressure reaches 200 mmHg) followed by 5 min. The deflation was performed continuously for 3 to 4 cycles as RIC protocol for ischemia treatment. Reperfusion profiles in STEMI depend on several patient-related factors. For example, age has a great influence on the regulatory ability of the cardiovascular system. Regardless of animal experiments or clinical trials, the protective effect of ischemic preconditioning and post-conditioning on older age is significantly reduced [27]. Although some studies have speculated that the effective thresholds for ischemic preconditioning and post-conditioning have increased for older cases, it is proposed to adopt higher intensity treatment measures to achieve effective cardio-protection, but some studies have shown that the intensified treatment measures still fail to overcome the decline in effectiveness. In addition, comorbidities also have an effect on the effects of RIC [27]. A variety of chronic diseases such as diabetes, obesity, hyperlipidemia, hypertension, and cardiac hypertrophy, etc. have negative effects on ischemic preconditioning and post-treatment [28, 29]. It is worth noting that sulphonyl urea anti-hyperglycemic agents impair the activation of ATP-sensitive potassium channels and thus affect the effect of preconditioning. The above comorbidities are also important risk factors for STEMI. There are many cases of STEMI combined with the above chronic diseases, which largely limits the clinical application of RIC.

There is a certain degree of heterogeneity between the studies in this meta-analysis. The main cause of heterogeneity may be the different limb ischemic conditioning schemes (e.g. number of times, ischemic duration, site of action, and pretreatment distance from PCI). The effect of ischemic treatment on the upper and lower extremities can also vary with the number of muscles in the upper and lower limbs. Of course, the heterogeneity may also be due to the age of the patient and whether it is due to chronic diseases such as diabetes and obesity status.What’s more, factors include time of ischemia, and TIMI flow grade at admission have been recognized as determinants of post-PCI myocardial injury. Initial coronary arteriography of the myocardial visible collateral circulation (TIMI flow grade > I) in STEMI patients is protective and may develop smaller infarct size, regardless of protection strategy [30]. Evaluating myocardial blood flow in certain emergencies is challenging, so the protective effect of these patients by intervention should be ruled out. In this analysis, only 3 trials [12, 15, 22]excluded patients with TIMI flow grade > I, and other trials included patients with TIMI flow grade II – III, which may influence the cardioprotective effect of RIC.

Several limitations of current analysis should be discussed. First, the RIC protocol was not uniform. 9 trials were performed on ischemic preconditioning, 4 on ischemic post-conditioning, and 1 on ischemic preconditioning and post-conditioning. Compared with ischemic preconditioning, ischemic post-conditioning is applied after ischemia has occurred and is more clinically operable. In view of the unpredictability of clinical myocardial ischemic events, compared with preconditioning, ischemic post-conditioning is more clinically operable after the onset of ischemia, so its application prospects for target organ protection are the most promising. However, ischemic preconditioning provides intervention in the time window of ischemia, does not prolong treatment time, is ethically accepted by people, and has good clinical feasibility. Future clinical trials should further verify the clinical effects of these two methods. Four studies chose the lower limb for remote treatment, and 9 studies chose the upper limb. Kolbenschlag et al. [31] conducted further studies on remote limb selection and found that the ischemic treatment of the upper and lower limbs can increase the blood flow of the skin, but the RIC of the upper limb can better trigger the protective effect. Second, the 14 trials included in this study are mostly small and / or single-center trials with varying quality. All RCTs in this meta-analysis used the different trial designs. Thus, this meta-analysis did not provide reliable results on the effects of RIC added to PCI for prevention of reperfusion injury in STEMI patients. In the future, relevant research needs to be further improved from the following aspects: increasing the sample size; proper random allocation and allocation of hidden programs; sufficient follow-up duration to observe the short-term and long-term effects; stratified analysis of RIC protocol, TIMI flow grade, and more comprehensive evaluation of the efficacy of RIC. Thirdly, due to the retrospective nature of all the included studies, bias still exists, which may impact the comparison of clinical outcomes.

## Conclusions

In this meta-analysis based on randomized clinical trials, RIC was associated with lower myocardial edema levels and myocardial salvage index and decreased the incidence of MACCE, while it had no significant beneficial effect on infarct size, TIMI flow grade III or LVEF. These findings suggest that RIC is a promising adjunctive treatment to PCI for theprevention of reperfusion injury in STEMI patients; however, multi-center, high-quality studies with a larger sample size, are required to verify its clinical efficacy.

## Abbreviations

RIC: remote ischemic conditioning
PCI: percutaneous coronary intervention
STEMI: ST-segment elevation myocardial infarction
LVEF: left ventricular ejection fraction
MACCEs: major adverse cardiac and cerebrovascular events
CI: confidence interval; OR, odds ratio
